# Pilot Study of an Internet Patient-Physician Communication Tool for Heart Failure Disease Management

**DOI:** 10.2196/jmir.7.1.e8

**Published:** 2005-03-26

**Authors:** Robert C Wu, Diego Delgado, Jeannine Costigan, Jane MacIver, Heather Ross

**Affiliations:** ^3^Cardiology and Heart Function ClinicSt. Mary's Regional Cardiac Care CentreKitchener ONCanada; ^2^Division of CardiologyUniversity Health NetworkToronto ONCanada; ^1^Division of General Internal MedicineUniversity Health NetworkToronto ONCanada

**Keywords:** Internet, disease management, congestive heart failure, physician-patient relations, communication

## Abstract

**Background:**

Internet disease management has the promise of improving care in patients with heart failure but evidence supporting its use is limited. We have designed a Heart Failure Internet Communication Tool (HFICT), allowing patients to enter messages for clinicians, as well as their daily symptoms, weight, blood pressure and heart rate. Clinicians review the information on the same day and provide feedback.

**Objective:**

This pilot study evaluated the feasibility and patients' acceptability of using the Internet to communicate with patients with symptomatic heart failure.

**Methods:**

Patients with symptomatic heart failure were instructed how to use the Internet communication tool. The primary outcome measure was the proportion of patients who used the system regularly by entering information on average at least once per week for at least 3 months. Secondary outcomes measures included safety and maintainability of the tool. We also conducted a content analysis of a subset of the patient and clinician messages entered into the comments field.

**Results:**

Between May 3, 1999 and November 1, 2002, 62 patients (mean age 48.7 years) were enrolled.. At 3 months 58 patients were alive and without a heart transplant. Of those, 26 patients (45%; 95% Confidence Interval, 0.33-0.58) continued using the system at 3 months. In 97% of all entries by participants weight was included; 68% of entries included blood pressure; and 71% of entries included heart rate. In 3386 entries out of all 5098 patient entries (66%), comments were entered. Functions that were not used included the tracking of diuretics, medications and treatment goals. The tool appeared to be safe and maintainable. Workload estimates for clinicians for entering a response to each patient's entry ranged from less than a minute to 5 minutes or longer for a detailed response. Patients sent 3386 comments to the Heart Function Clinic. Based on the content analysis of 100 patient entries, the following major categories of communication were identified: patient information; patient symptoms; patient questions regarding their condition; patient coordinating own care; social responses. The number of comments decreased over time for both patients and clinicians.

**Conclusion:**

While the majority of patients discontinued use, 45% of the patients used the system and continued to use it on average for 1.5 years. An Internet tool is a feasible method of communication in a substantial proportion of patients with heart failure. Further study is required to determine whether clinical outcomes, such as quality of life or frequency of hospitalization, are improved.

## Introduction

Intensive management of patients with heart failure improves the quality of care, has a positive impact on quality of life, and reduces readmissions [1-3] through frequent monitoring, detailed assessment, optimization of medications, and education. Nevertheless, even with current optimal management, quality of life is still poor in patients with symptomatic heart failure. Thus improving quality of life remains a major goal in the treatment of heart failure [4].

The Internet has shown promise in the care of chronic disease particularly in obesity. Patients who received Internet behavioural counselling lost more weight after 1 year than patients randomized to receive only Internet educational information [5,6]. There have also been pilot studies of Internet interventions in cardiac care including cardiac transplantation [7] and heart failure [8]. A randomized controlled trial in heart failure, which focused primarily on providing patients with Internet access to their medical record, failed to show a difference in quality of life [8]. It was, however, well received by patients, and the patients believed the Internet could in theory deliver benefits including improved education, coordination of care, and self-care [9].

Similar to Internet management of diabetes [10], an Internet disease management tool could improve the care of patients with a chronic disease such as heart failure in the following ways: improve monitoring of patients, provide a method for clinicians to educate patients about their condition, provide individualized feedback and reassurance, and provide a framework for patients to self-manage their disease.

We have designed a tool to achieve these goals. The Heart Failure Internet Communication Tool (HFICT) was developed to enable electronic communication between clinicians and patients with heart failure to help manage their disease. Patients can enter parameters that are important to monitor in heart failure: symptoms, weight, blood pressure and heart rate. Patients would enter this information usually on a daily basis to allow the clinicians to monitor them closely. A clinician reviews the information on the same day and provides feedback, including educational messages, reassurance or suggesting a change in therapy. Our overall goal was to improve care by improving communication and better educating patients on how to manage their condition. Preliminary findings with 16 patients who used the tool showed a trend towards improved quality of life as well as high satisfaction levels [11].

A randomized controlled trial of Internet communication is needed to determine whether such an Internet disease management tool can improve care delivery and outcomes in individuals with congestive heart failure. In preparation for such a trial, we conducted a pilot study to determine whether patients would use such a tool over a sustained period of time, and to evaluate the safety and maintainability of such a tool.

## Methods

This study was a prospective observational cohort study. The study was conducted in the Heart Function Clinic, Toronto General Hospital, University Health Network, Toronto, Ontario, Canada. The Heart Function Clinic is a multidisciplinary clinic receiving referrals for patients with complex heart failure.

### Participants

As this was a pilot study, a convenience sample was used consisting of patients enrolled through the clinic over a period of 3 years (May 3, 1999 and November 1, 2002).

Eligibility criteria included new referral to the clinic with a diagnosis of heart failure, New York Heart Association (NYHA) functional class III or IV, and a left ventricular ejection fraction less than or equal to 30%. Exclusion criteria were lack of Internet access, inability to obtain their own body weights at home, and expected survival less than 3 months. Internet access was defined as having personal access or access through a trusted family member or friend.

### Design

The Institutional Review Board at the University Health Network approved the protocol. Patients who were eligible based on the inclusion and exclusion criteria were given the option of using the Heart Failure Internet Communication Tool. Patients were instructed that if they declined to participate, they would still receive the usual standard of care provided by the Heart Function Clinic. Written informed consent was obtained from all participants. Following completion of baseline measurements, all consenting participants were instructed in the use of the HFICT. Measures were taken to protect confidentiality according to guidelines in effect at the time [12].

During the informed consent process, patients were told that a possible benefit was that the system could improve patient-clinician communication, which in turn could improve their care and possibly their quality of life. They were also told that risks included possible compromised confidentiality of health information when transmitted over the Internet, as well as possible delays in obtaining care if the Internet was used for urgent communication.

### Internet Intervention

**Figure 1:**
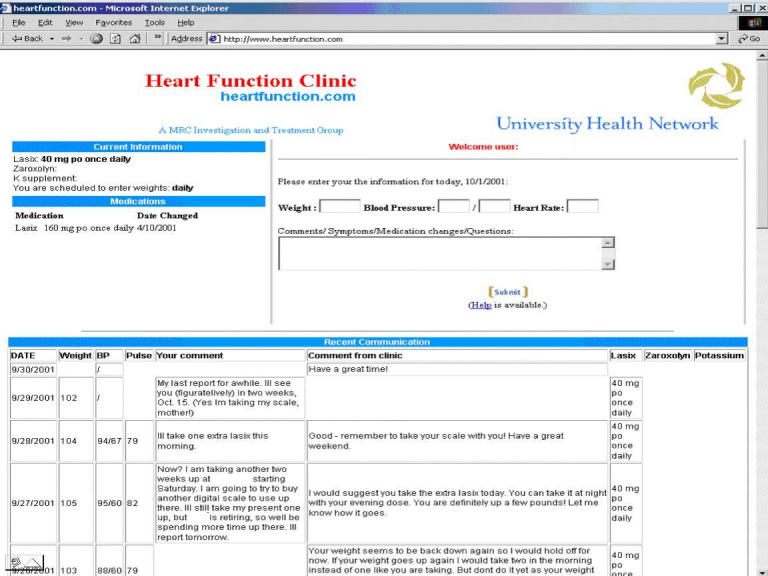
Screenshot of patient's data entry screen, with recent communication displayed

Participants were instructed on how to enter their weight, blood pressure, heart rate as well as any symptoms into the HFICT. Regular clinic practice is to prescribe blood pressure monitors for patients for whom it is important to monitor blood pressure. Based on the severity of their heart failure, participants were told how often to enter their information (from daily to once a week) Figure 1. Clinicians (nurse-practitioner or cardiologists) reviewed and responded to patients' entries using the online messaging tool each weekday. Clinicians answered questions, educated, provided reassurance, and changed medications when necessary.

As this was a method of nonurgent communication, patients were instructed at enrollment not to use the website for urgent communication. For urgent contact, patients were instructed to telephone the clinic, telephone their family physician or go to the Emergency Room as appropriate. These instructions were also displayed on the website. If patients tried to use the website for urgent communication, they would be instructed to seek medical attention promptly and were reminded that the Internet communication tool was not a method of urgent communication.

### Baseline Patient Measures

The following baseline characteristics were obtained from the patient at enrollment: age, gender, medications, NYHA class, etiology of heart failure, left ventricular function and co-morbid conditions.

### Primary Outcome

The primary outcome was the usage of the HFICT. Usage of the HFICT was defined as regular interaction (at least once per week on average) with the HFICT for a minimum of 3 months. While regular contact (daily or weekly) was desired, participants could miss a week for valid reasons such as vacation or hospitalization. In order to be considered users, patients did not need to log in every week, but they did need to enter information into the HFICT on average once per week. Patients were also required to continue entering information for at least 3 months to be considered users. The follow-up period of 3 months is that used in previous studies of telephone-based interventions [13] and home-based interventions [14]. Regular interaction (at least once per week on average) is assumed to be necessary to derive benefits, such as improved knowledge, monitoring and self-care skills, from the tool. Patients who died or required transplantation within 3 months were excluded from this outcome analysis.

### Secondary Outcomes

A number of secondary endpoints assessing safety, effectiveness, security and maintainability further determined the feasibility of the HFICT. While we hope that this tool will improve quality of care, we recognize that this new intervention could be unsafe. If patients or clinicians rely on this tool too much and do not get appropriate follow-up, an increase in hospitalizations or deaths could occur. In order to evaluate the safety of the system, the following outcomes were monitored:

- unplanned hospitalizations
- unplanned hospitalizations due to heart failure
- planned hospitalizations (admissions that were scheduled in advance, such as pacemaker insertion or tailored inotropic therapy)
- mortality
- cardiac transplantation
- security breaches including incorrect logins and reported breaches as reported by participants, health care providers and administrators of the site

Since participants had the option of continuing to use the system beyond 3 months, we followed all enrolled participants (users and nonusers) for the mortality, hospitalization and transplantation endpoints until the study end date.

Maintainability was assessed by estimating workload on the clinicians as well as the training requirements of clinicians.

### Qualitative Message Content Analysis

In order to better understand the nature of communication with the HFICT, a qualitative content analysis of patient messages and clinician responses was performed. A random 100 entries from the participants and 100 entries from the clinicians were reviewed to look for common themes. This coding structure was then applied to all remaining comments to quantify the content of communication. As this was a pilot study, there was only one coder, the primary investigator (RW).

### Statistical Analysis

Descriptive statistics were expressed as mean ± standard deviation. Patients who used the HFICT were compared to patients who did not use the system. Associations were tested using the chi-square statistic for categorical variables and unpaired *t* tests for continuous variables. For all analyses, an alpha error of ± .05 was considered significant.

As the primary outcome was a proportion, a confidence interval was calculated for this proportion.

For the analysis of the safety data, patients who used the intervention were grouped and were compared to the patients who were enrolled but who did not use the HFICT. The endpoints such as mortality and transplant were calculated as proportions and were compared by chi-square analyses. For the end points of hospitalizations, mean hospitalizations per patient were calculated. For these continuous variables, an unpaired *t* test for association was performed.

## Results

Between May 3, 1999 and November 1, 2002, 62 patients were enrolled. All patients were followed until March 2003, with a total patient follow-up of 109 patient-years. During the total follow up period, 11 patients died , giving an annualized mortality rate of 10.1%. 

The baseline characteristics for the 62 patients are listed in Table 1. Those who used the HFICT were older, and more likely to be female, to have idiopathic dilated cardiomyopathy, to have a worse functional class, and to be on proven medications for heart failure such as Angiotensin Converting Enzyme (ACE) inhibitors and beta-blockers. These trends were not statistically significant.

**Table 1:** Baseline characteristics

**Variable**	**All(N=62)**	Users^*^(n=26)	**Nonusers(n=36)**	***P* value**
Age	48.7 ± 13.0	52.4 ± 12.1	46.1 ± 13.2	.06
Men (%)	69%	61.5%	75%	.26
Co-morbidities Diabetes Coronary disease Number of co-morbidities (mean)	19.4% 21.0% 1.7±1.5	19.2% 19.2% 1.8±1.5	19.4% 22.2% 1.6±1.4	.99 .77 .60
NYHA class IV	4 (6%)	3 (12%)	1 (3%)	.20
Left ventricular grade	3.4±0.6	3.4±0.6	3.5±0.5	.56
Etiology of heart failure Idiopathic dilated Ischemic Other	43.5% 25.8% 30.6%	46.2% 23.1% 30.8%	41.7% 27.8% 30.6%	.72 .68 .99
Medications ACE inhibitor ARB^†^ Beta blocker Loop diuretic Spironolactone Digoxin	80.6% 14.5% 77.4% 75.8% 43.5% 69.4%	84.6% 15.4% 84.6% 69.2% 42.3% 73.1%	77.8% 13.9% 72.2% 80.6% 44.4% 66.7%	.50 .87 .25 .30 .87 .59
Baseline LHFQ^‡^ (n)^§^	57.5 (43)	62.2 (22)	52.5 (21)	.10

^*^ Defined as a participant who used the system for at least 3 months, on average once per week

^†^ ARBAngiotensin II receptor blocker

^‡^ Minnesota Living with Heart Failure Questionnaire [15]

^§^ Baseline LHFQ collection was incomplete. The number of subjects who completed the baseline questionnaire is listed in parenthesis.

### Primary Outcome: Use of the HFICT

Of the 62 patients who were enrolled, 3 out of the 11 patients who died during the follow-up period passed away within 3 months after enrollment and 1 had a heart transplant, thus at 3 months we collected usage data from 58 patients. Of these 58 patients, 26 used the system for at least 3 months on average once per week (45%; 95% Confidence Interval [CI], 0.33-0.58). Of the nonusers, 23 patients were enrolled but never logged in; 14 patients logged on at least once but did not continue for 3 months. There were 3 patients who died within 3 months of enrollment, and 1 patient who underwent heart transplantation 1 month after enrollment. After 12 months, only 16 patients continued to use the system, all others stopped using the system. Of these, 8 continued to use the system at 2 years and 4 continued to used the system after 3 years.

With respect to the participants' use of individual components of the system, certain information was entered more frequently than others. In 97% of all entries by participants weight was included; 68% of entries included blood pressure; and 71% of entries included heart rate. In 3386 entries out of all 5098 patient entries (66%), comments were entered. Functions that were not used included the tracking of diuretics, medications and treatment goals. Diuretic changes were instead documented in the “Comments” section. Medications were initially entered at enrollment but were not kept up to date for the majority of patients (79%). Patient-specific goals such as the target weight and beta-blocker titration were not entered at all by clinicians. Usage data are shown in Table 2.

**Table 2:** HFICT usage data for those defined as ‘users'

	**Mean (SD)**	**Range**
Number of entries per user	191 (175.0)	27-636
Number of months of Internet follow-up	18.8 (12.0)	5.8-42.0

### Secondary Outcomes

#### Safety

Table 3 lists the different safety endpoints in the HFICT, comparing users to nonusers. There was no excess of death or transplants in the user group. Mortality in the nonuser group was higher but not statistically significant (user 11.5%, nonuser 22.2%, *P*= .28). Transplantation was higher in the user group but again not statistically significant (user 15.4%, nonuser 5.6%, *P*=.20).

**Table 3:** Comparison of two groups for safety endpoints (during total follow-up period until March 2003)

	**Users (n=26)**	**Nonusers (n=36)**	***P* Value**
Deaths – n (%)	3 (11.5%)	8 (22.2%)	.28
Transplant – n (%)	4 (15.4%)	2 (5.6%)	.20
Hospitalizations			
Total – n (mean per pt)	28 (1.08)	18 (0.50)	.10
Planned – n (mean per pt)	8 (0.31)	3 (0.08)	.04
Unplanned – n (mean per pt)	20 (0.77)	15 (0.42)	.26
Unplanned due to heart failure – n (mean per pt)	15 (0.58)	13 (0.36)	.40

There were more hospitalizations in the user group (mean hospitalizations: per user 1.08, per nonuser 0.50, *P*=.10).This was predominantly due to the statistically significant difference (*P*=.04) in the planned hospitalizations for procedures such as pacemakers, implantable cardiac defibrillators or tailored inotropic therapy.

The HFICT communications and the charts were reviewed and no errors attributable to the communication process were detected. Rather, the recurrent admissions were felt to be appropriate for the severity of heart failure.

Of the 11 patients who died, 4 died within 1 month of entering information. To determine whether the use of HFICT contributed to these deaths, the characteristics of these patients were examined further. As Table 3 shows, none of the 4 patients appeared unstable by weight change, vital signs or comments at the time of their last entry. Only 1 of these patients was defined as a user and had been using the HFICT for almost 1 year before a death that was not heart failure-related.

There were no reports or indications of lapses in security or confidentiality.

**Table 4:** Characteristics of patients who died within 1 month of having recently used the HFICT

**User**	**Time on HFICT**	**Number of Entries**	**Recent Weight Trend**	**Recent Symptoms**	**Heart Failure Related Cause of Death**
Yes	310 days	112	Stable	None	No
No	3 days	1	^*^	None	Yes
No	29 days	9	Stable	None	Presumed
No	70 days	33	Stable	None	Presumed

^*^ Unable to determine due to only one entry on the system

#### Maintainability

Workload estimates for clinicians for entering a response to each patient's entry ranged from less than a minute to 5 minutes or longer for a detailed response. If further information was required, such as determining the side effects of a new medication or verifying details with a cardiologist, a response could take up to a half hour. Clinician monitoring and entries were done predominately by nurse-practitioners (98.3% of entries) with the remainder by cardiologists.

Website training was given to 3 cardiologists and 3 nurse-practitioners. All were able to use the website without problems after a half-hour training session.

Technical costs of the system included development time (approximately 200 hours) and system support (2 hours per month). Hardware and software costs were minimal as shared resources were used.

There were 5 occurrence when the system was not available, and these happened in the first 2 years of the pilot. Of these 3 occurred in the first 6 months of the study, resulting in 3 downtimes of several days each. The causes of these problems were corrected and no further downtimes were experienced in the final 2 years. After any downtime, many participants communicated the importance of keeping the website always available.

#### Analysis of Communication Content

Over the entire study period, patients sent 3386 comments to the Heart Function Clinic. Based on the qualitative review of 100 entries, the following major categories of communication were identified:

- patient information (eg, blood glucose, outside laboratory values, description of visits with family physician)
- patient symptoms (eg, shortness of breath, dizziness, ankle swelling, chest pain)
- patient questions regarding their condition (eg, how much diuretic to take, whether symptoms are side effects of medications)
- patient coordinating own care (eg, organizing next clinic appointment, arranging other tests such as angiogram)
- social responses (eg, statements regarding weather)

**Figure 2:**
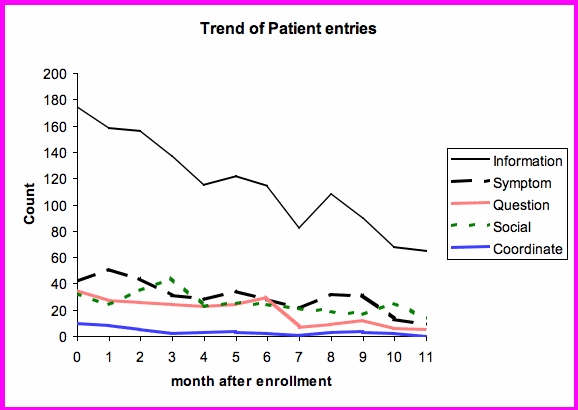
Trend of patient entries into the comments field over 12 months, adjusted for patient drop-out

Figure 2 shows the trend of patient communication over time after enrollment. The number of comments decreased over time. Most communication consisted of patients providing information. Symptoms, questions, and social communications were all entered with similar frequency, declining over time. A small proportion of comments were used to coordinate care.

**Figure 3:**
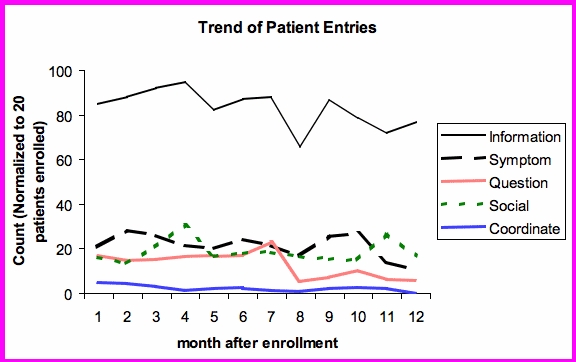
Trend of patient entries over 12 months, adjusted for patient drop-out

In order to reduce the effect of participant dropout, the count of entries per month were normalized to 16 patients enrolled (the number of patients enrolled at 12 months). Figure 3 shows that for an average participant who continues to use the system, most of the categories are stable except for questions regarding their condition.

Over the entire study period, there were 3219 responses from the clinicians. From the qualitative review of 100 entries, the following categories of communication were identified:

- education about heart failure (eg, explaining weight gain and salt intake)
- reassurance regarding their heart failure management (eg, encouraging that they are doing well managing their heart failure)
- questions on their symptoms (eg, asking if they have increased their salty food intake, or the nature of their chest pain)
- instructions to change their management (eg, increase diuretic, seek medical attention)
- social responses

**Figure 4:**
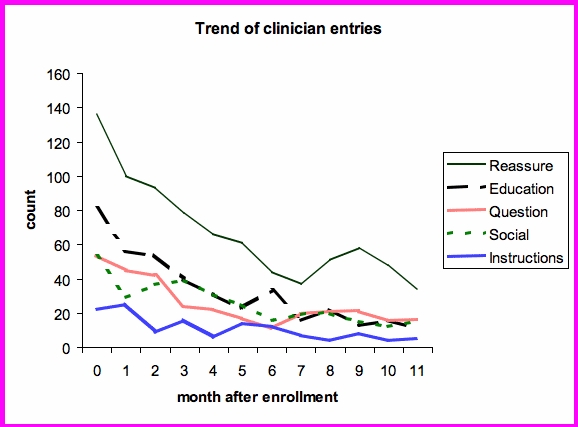
Trend of clinician responses over 12 months, adjusted for patient drop-out

Similar to patient comments, the rest of the clinician responses were coded to these categories. Figure 4 shows the trend of clinician responses over time after enrollment. Predominantly, most communication dealt with reassurance. Initially, education was second in frequency but declined over 6 months. Questions, social communications, and instructions were all entered with about the same frequency, declining over time.

**Figure 5:**
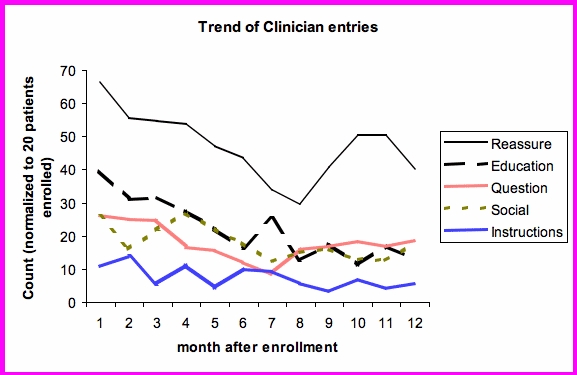
Trend of clinician responses over 12 months, adjusted for patient drop-out

Again, the effect of dropout was taken into account by normalizing the entries to 16 patients enrolled. As can be seen in Figure 5, educational messages and reassurance decreased whereas other categories like social interactions and instructions regarding care remained about the same level over the 12 months.

#### Discussion

In this pilot study of a Heart Failure Internet Communication Tool, we evaluated whether patients with significant morbidity would communicate using the Internet with their clinicians. Our study population consisted of outpatients with heart failure who had significant symptoms and left ventricular dysfunction. We found that 45% of patients used the system for at least 3 months.

While it is encouraging that almost half of the enrolled patients used the system, it is important to determine why more patients did not use the system. This is crucial for the success of this system as well as other Internet communication tools for chronic disease management. Likely reasons for not using the system include lack of perceived benefit, and system usability issues. Patients might not use the system if there is a lack of interest in their condition or if they are already satisfied with their knowledge level regarding self-care. Although we enrolled only patients who had access to the Internet, this does not mean that they were sufficiently familiar with it or that they are comfortable using it to communicate about their condition. Finally, there is a significant time commitment even for those who are interested, likely up to 10 minutes per day to collect and enter information.

Patients decide to use the system because they expect that they would feel they would derive some benefit. Those patients most likely to benefit are those with significant morbidity from heart failure. Our data suggests that those who did use the system had more symptoms since the group that used the system had a worse NYHA functional class and a worse quality-of-life at baseline.

Finally, there were system issues that may have decreased compliance. System-specific issues, which were encountered, included several server crashes and software problems. The system underwent gradual improvements, and early problems were eventually resolved. However, system issues are unlikely to be a major factor in nonusage, as the majority of nonusers (64%) did not even login once.

The usage rate of 45% is comparable to other Internet communication tools. In a study of patients who registered for electronic messaging with their primary care physician, 47% used the system 3 or more times [16], while a previous study of heart failure patients found that approximately 35% continued to access their electronic record through the Internet [8].

The HFICT appeared safe to use, but there was a trend towards more hospitalizations in the user group. While it is hoped that the HFICT would decrease resource utilization, in fact, an increase in hospitalizations may be medically appropriate and indicate high quality care. In several instances, there were instructions by the clinicians via the HFICT to the patients to go to the Emergency Room for symptoms such as ”dizzy” spells that patients did not feel were significant or did not attribute to their heart failure. It is likely that if they had not been using the system they would not have sought medical attention. Interestingly, a recent study which provided heart failure patients access to their medical records was also associated with an increase in Emergency Room visits [8]. Further study is necessary to determine if there is an increase in unwarranted, unplanned hospitalizations.

Our study found that a low-cost solution was acceptable in a substantial proportion of patients who had significant morbidity. System usage has been defined as a measure of success for nonmandatory information systems [17]. With our system, approximately half of the patients used the system and continued to use it on average for 1.5 years, entering information on average 191 times per person.

We found that patients used the tool primarily to communicate general information but also used the tool for asking specific questions and for social interaction. Reassurance and education were the important parts of the communication from the clinicians. This may support the hypothesis that patients are learning to manage their heart failure through this tool.

The limitations to this study include small sample size, lack of a control group and possible selection bias. Enrollment was low and not tracked. Qualitative studies, such as surveys and focus groups of nonusers would also clarify reasons for not using the tool. Selection bias and limited power make it impossible to draw definitive conclusions about the effects of HFICT on morbidity and mortality. Finally, the study population was a select group of heart failure patients, those referred to a specialized clinic including a substantial proportion of pre-transplant patients. Thus, our results may not be generalizable to patients seen in internal medicine clinics, general cardiology clinics, or possibly even other heart failure clinics. Nevertheless, patients attending tertiary heart failure clinics have significant morbidity and mortality, and interventions that improve quality of life and are cost-effective are still worthwhile even in this ”select” population. Further study would be required in other populations including those seen in other settings to see if the use of a HFICT is generalizable to the majority of patients with heart failure.

In conclusion, we found that 45% of patients with heart failure in our clinic would use an Internet disease management tool. Furthermore, we found it to be safe and maintainable. The HFICT appears to be a feasible method of helping to manage patients with a chronic disease such as heart failure. Further study is required to determine if it improves care and outcomes.

## Abbreviations

HFICT: Heart Failure Internet Communication Tool
HYHA: New York Heart Association
